# In-vitro and in-silico evidence for oxidative stress as drivers for RDW

**DOI:** 10.1038/s41598-023-36514-5

**Published:** 2023-06-07

**Authors:** Huibert-Jan Joosse, Brigitte A. van Oirschot, Sander A. A. Kooijmans, Imo E. Hoefer, Richard A. H. van Wijk, Albert Huisman, Wouter W. van Solinge, Saskia Haitjema

**Affiliations:** Central Diagnostic Laboratory, University Medical Center Utrecht, Utrecht University, Utrecht, The Netherlands

**Keywords:** Data mining, Machine learning, Diagnostic markers, Erythropoiesis

## Abstract

Red blood cell distribution width (RDW) is a biomarker associated with a variety of clinical outcomes. While anemia and subclinical inflammation have been posed as underlying pathophysiology, it is unclear what mechanisms underlie these assocations. Hence, we aimed to unravel the mechanisms in silico using a large clinical dataset and validate our findings in vitro. We retrieved complete blood counts (CBC) from 1,403,663 measurements from the Utrecht Patient Oriented Database, to model RDW using gradient boosting regression. We performed (sex-stratified) analyses in patients with anemia, patients younger/older than 50 and validation across platforms and care settings. We then validated our hypothesis regarding oxidative stress using an in vitro approach. Only percentage microcytic (pMIC) and macrocytic (pMAC) erythrocytes and mean corpuscular volume were most important in modelling RDW (RMSE = 0.40, R^2^ = 0.96). Subgroup analyses and validation confirmed our findings. In vitro induction of oxidative stress underscored our results, namely increased RDW and decreased erythrocyte volume, yet no vesiculation was observed. We found that erythrocyte size, especially pMIC, is most informative in predicting RDW, but no role for anemia or inflammation. Oxidative stress affecting the size of the erythrocytes may play a role in the association between RDW and clinical outcomes.

## Introduction

Red Blood Cell (or: Erythroycte) Distribution Width (RDW) is reported extensively as a biomarker predictive for future deterioration of illness. RDW is a measure of the distribution of the volume of erythrocytes, derived from the mean corpuscular volume (MCV) by dividing the standard deviation of erythrocyte volume (RBCV) by the mean volume (MCV), and multiplying this by 100 to present the final number as coefficient of variation in percentages (i.e.: (σRBCV/MCV) × 100)^1^. A higher RDW has been associated with a large variety of adverse clinical outcomes, including heart failure, cancer treatment outcomes, postoperative sepsis development, improving ICU scoring systems, long term outcomes in sepsis, trauma, (hip) fractures in the elderly, and mortality^2–11^.

Yet, it is still unclear how RDW may reflect the underlying pathophysiology of this plethora of clinical outcomes. In literature, MCV itself is not considered the underlying culprit. Some studies point towards microcytic anemia via malnutrition which increases anisocytosis^2^. Others point towards a possible role of inflammation^12,13^, which can lead to disruption of erythropoiesis and premature erythrocyte destruction, resulting in microcytic anemia^14^. The only way to test these hypotheses is by using a very large dataset with available RDW as well as accompanying blood cell characteristics to study the role of anemia, inflammation and other possible pathophysiological mechanisms.

In the UMC Utrecht, Utrecht, the Netherlands, a detailed blood panel of 79 hematological parameters, including complete blood count (CBC), erythrocyte measurements, leukocyte differentiation and ‘research-only’ values regarding leukocytes, erythrocytes and platelets, is measured by hematology analyzers in routine clinical care and stored in the Utrecht Patient Oriented Database (UPOD)^15^. This way, RDW and accompanying hematological values are available including research-only values that are not used in clinical practice, but are measured as a result of hematological analysis in routine care.

In this study, using a homogenous data set with over 1.4 million measurements from the UPOD, we aimed to unravel the pathophysiological mechanisms underlying the association between RDW and clinical outcomes, specifically anemia and inflammation, by creating a machine learning model to find associations with regards to RDW, and further study these in silico findings in an in vitro study.

## Materials and methods

### Database

For this study data from the Utrecht Patient Oriented Database (UPOD) were used^15^. In brief, UPOD is an infrastructure of relational databases comprising data on patient characteristics, hospital discharge diagnoses, medical procedures, medication orders and laboratory tests for over 2.5 million patients treated at in and outpatient clinics at the University Medical Center Utrecht (UMC Utrecht), Utrecht, the Netherlands, since 2004. UPOD data acquisition and management is in accordance with current regulations concerning privacy and ethics including the General Data Protection Regulation (GDPR).

In the UPOD 79 hematological characteristics are stored as a result of measurements during routine care using the Cell-Dyn Sapphire hematology analyzer (Abbott Diagnostics, Abbott, Santa Clara, California, USA). These characteristics encompass erythrocytes, leukocytes, platelets, and reticulocytes, as well as characteristics that are directly derived from the raw measurements i.e., characteristics on axial light loss (ALL), intermediate angle scatter (IAS), polarized and depolarized side scatter (PSS and DSS respectively), and fluorescence of erythrocytes and leukocytes, as measured by the FL1 and FL3 channels respectively. A full description of all blood cell characteristics can be found in supplementary Table 1.

### Study population

**Figure 1:**
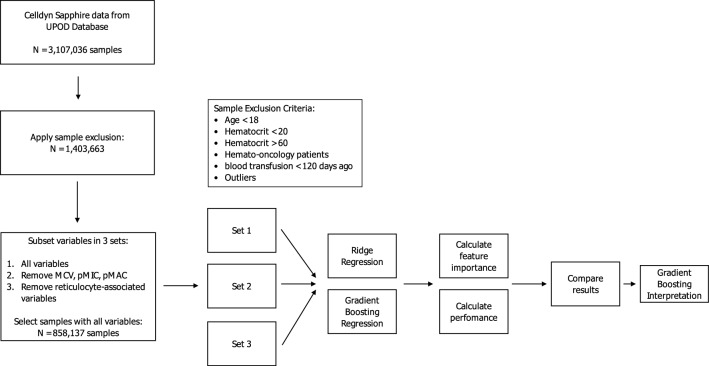
Flowchart of the discovery analysis steps followed in this project.

As we wanted to study the general determinants for RDW, we extracted a homogeneous discovery dataset and excluded the following patients (Fig. 1): patients aged < 18, hemato-oncology patients and measurements with a hematocrit value < 0.20 or > 0.60 (blood cancers, and bone marrow disorders). Additionally, patients that received blood transfusion < 120 days prior to their lab measurement were excluded because their erythrocytes do not reflect true erythroid homeostasis. To further ensure a homogenous discovery data set and exclude remaining hemato-oncology patients, outliers for the white blood cell (> 50 × 10^9/L), eosinophil (> 5 × 10^9/L), and basophil (> 5 × 10^9/L) counts, white blood cell viability factor (< 0.8), and the coefficients of variance of the platelet complexity (> 100 AU) and lobularity (> 100 AU) were excluded. We allowed for multiple observations per patient meaning that we selected all available measurements for each patient, not taking into account the time between measurements or amount of measurements per patient. The study was conducted in accordance with the declaration of Helsinki. The institutional review board (Medical Research Ethics Comittee NedMec) waived the need for informed consent (IRB number 18/130) as only pseudonymized data were used for a large patient sample. This study was not subject to the Human Subjects Act (in Dutch: Wet Medisch-Wetenschappelijk onderzoek met mensen, WMO) and we therefore obtained a waiver for study approval from the institutional review board (Medical Research Ethics Comittee NedMec).

### Blood cell characteristics

We used all available hematological characteristics, yet excluded highly correlating variables and variables reflecting percentages (Supplementary Figs. 1 and 2). To study the role of (chronic) inflammation on RDW, we included counts of leukocytes, neutrophil count, monocyte count, lymphocyte count, eosinophil count and basophil count, as well as variables reflecting size, complexity, and viability of these cell types. As modelling outcome, we used RDW as calculated by the Abbott algorithm, which uses the erythrocyte volume distribution at 50% of the peak height^16^.

For modelling, the blood cell data was further subsetted to be able to compare different sets of predictors for RDW. These subsets were built as follows: all of the variables were used for a full estimation of all relevant blood cell characteristics (Set 1). For Set 2 MCV, percentage microcytic erythrocytes (pMIC), and the percentage macrocytic erythrocytes (pMAC) were omitted from set 1 because they are obvious drivers of RDW as they are derivatives of MCV which in turn is used to calculate RDW. In Set 3 all parameters linked to reticulocyte counts were omitted from set 2, because these variables show the level of maturity for erythrocytes which in turn influences RDW (i.e., immature erythrocytes have a larger size, thus influencing the RDW).

### Clinical chemistry characteristics

Folic acid, vitamin B12, iron and ferritin were measured according to standard diagnostic procedures on routine analyzers (AU5800 and Dxi, Beckman Coulter Inc., Brea CA, USA).

### Descriptive analyses

First, we scrutinized RDW by descriptive analytics. Because associations are known between sex and age and RDW^12,17^, we tried to replicate those. All variables are presented as means +/− SD for normally distributed variables and medians +/− IQR for non-normally distributed variables. We modelled the effect of RDW on age by linear regression and studied differences between the sexes by the Mann–Whitney U test.

### Modelling approaches

In order to analyze the association of other blood markers with RDW values we used two different modelling approaches (summarized in Fig. 1). First, we used a more classic approach using Ridge regression^18^. Additionally, to capture non-linear associations, a gradient boosting (GB) regression method was used^19^. Each analysis method was used for modelling RDW with the use of the three sets of determinants (Set 1–3), and for each of these sets, a tenfold cross-validation was carried out. We assessed feature importance of the blood cell characteristics in predicting RDW in the three sets by using shapley values (calculated per fold and averaged), indicating importance of a variable in a specific model in predicting a certain outcome. The resulting importance for a feature is the mean difference between the model prediction with and without said variable^20^. For computational reasons the feature importances for the GB models were calculated on at most 10,000 random samples per fold. To assess the resulting models, the performance was measured using the Root Mean Squared Error (RMSE, lower score is better) and the R^2^ score (higher score is better). Considering the relatively poor performance of the Ridge regression models and the aim of this research, we followed-up on the GB regression models only, as they modelled RDW more accurately.

### Subgroup analyses

To further unravel the found effects, we performed sensitivity analyses inour data for different subgroups by selecting these subgroups as follows: anemic patients (Hb < 13.9 g/dL for men, < 11.9 g/dL for women), people with microcytic and macrocytic anemia in subgroups of patients with low iron (< 8umol/L for men, < 5 umol/L for women), low ferritin (< 25 ug/L for men, < 20 ug/L for women), low folic acid (< 6.8 nmol/L) or low vitamin B12 levels (< 130 pmol/L). In addition, we performed a subgroup analysis on patients with age > 40, patients < 40, and per sex. Lastly, different subgroups along the hemoglobin distribution curve were analyzed (low: < 15.5 g/dL for men, < 13.7 g/dL for women, normal: 13.9–17.2 g/dL for men, 11.9–15.5 g/dL for women, high: 15.5–17.2 g/dL for men, 13.7–15.5 g/dL for women) to study the possible effects of subclinical anemia. For each of these groups, we followed the same approach for the overall population i.e., we developed both a ridge regression and GB regression model, and assessed their performance in these subgroups.

### In-silico validation studies

In order to assess the generalizability of our model, we selected data from the Abbott Cell-Dyn Sapphire as well as the new Abbott Cell-Dyn Alinity hq (Abbott Diagnostics, Abbott, Santa Clara, California, USA) for the same measurements (December 2021). These measurements were measured in parallel as part of a comparison study between the Cell-Dyn Sapphire and Alinity analyzers. We selected the overlapping variables and applied the same patient filters that corresponded with our selection criteria in the discovery data set.

Additionally, to validate our findings in different care settings, we used a Cell-Dyn Sapphire data set containing samples measured in primary and secondary care only. We selected data based on the same inclusion criteria in our original data set, and excluded the hemato-oncology patients based on the same blood cell characteristics (blood transfusion data were unavailable).

### Software

All analyses except for the Mann–Whitney U and Kruskal–Wallis test (R version 3.6.2) were carried out using Python 3.7. Ridge regression was carried out using *scikit-learn* package version 0.22.1 and the GB regression was carried out using the *xgboost* package version 0.90. Feature importances were calculated using the *SHAP* package version 0.34.0.

### Erythrocyte isolation, stimulation and analysis

To follow up on our findings in silico and hypothesis concerning the role of oxidative stress on RDW*,* we performed in vitro induction of oxidative stress in triplicate, for which blood from healthy volunteers was collected in K_2_EDTA tubes (approved by the institutional review board). Erythrocytes were isolated using α-cellulose columns as previously reported^21^, washed with saline and resuspended in Ringer buffer (32 mM HEPES, 125 mM NaCl, 5 mM KCl, 1 mM MgSO_4_, 1 mM CaCl_2_, 5 mM glucose, pH 7.4) at a final hematocrit of 40%. erythrocytes were stimulated with tert-Butyl Hydroxyperoxide (5 or 7.5 mM) (tBHP, Luperox, Aldrich 458,139) or Diamide (5 or 10 mM Diamide) (Sigma D3648) for 30 min, on a rotator at room temperature. Alternatively, the cells were stimulated with 1-Acetyl-2-phenylhydrazine (5 mg/mL) (PHZ, Sigma A4626) for 1 h at 37 °C.

After treatment, the cells were analyzed using a Cell-Dyn Sapphire hematology analyzer. Additionally, deformability of erythrocytes was analyzed using the osmoscan module on the Laser Optical Rotational Red Cell Analyzer (Lorrca, RR Mechatronics, Zwaag, The Netherlands)^22^.

### Nanoparticle tracking analysis

For vesiculation experiments, after treatment, cells were pelleted at 1000 g for 10 min at room temperature. Supernatants were transferred to new tubes, diluted fivefold with phosphate buffered saline (PBS), and centrifuged again at 1000 g for 10 min at 4 °C. Aliquots of the supernatant were diluted 1:20 with PBS and analyzed using a NanoSight NS500 system equipped with an LM14 405 nm violet laser unit (Malvern Instruments, Worcestershire, UK). For each sample, 5 movies of 30 s were recorded at camera level 15. Analysis of particle concentration was performed using NTA 3.4 software with detection threshold set at 7.

## Results

After filtering, 1,403,663 observations (214,315 patients) remained (Fig. 1). Mean age was 55.26, 51% observations from male patients. Median RDW was 12.62% (IQR 11.84–14.06%).

### RDW differs between ages, sexes and birth months

To confirm previous findings, we replicated the differences in RDW over age (β = 0.018 RDW increase per year, Supplementary Fig. 3), and sexes (median men: 12.67% (IQR: 11.90–14.11%), women: 12.56% (IQR 11.78–13.99%) (W = 2.57 × 10^11^, *p* < 0.001)). We replicated the sex-differences in the population below 40 (W = 1.25 × 10^10^, *p* = 0.041) as well as above 40 (W = 1.44 × 10^11^, *p* < 0.001) years of age.

### RDW is best explained by erythrocyte characteristics

**Figure 2:**
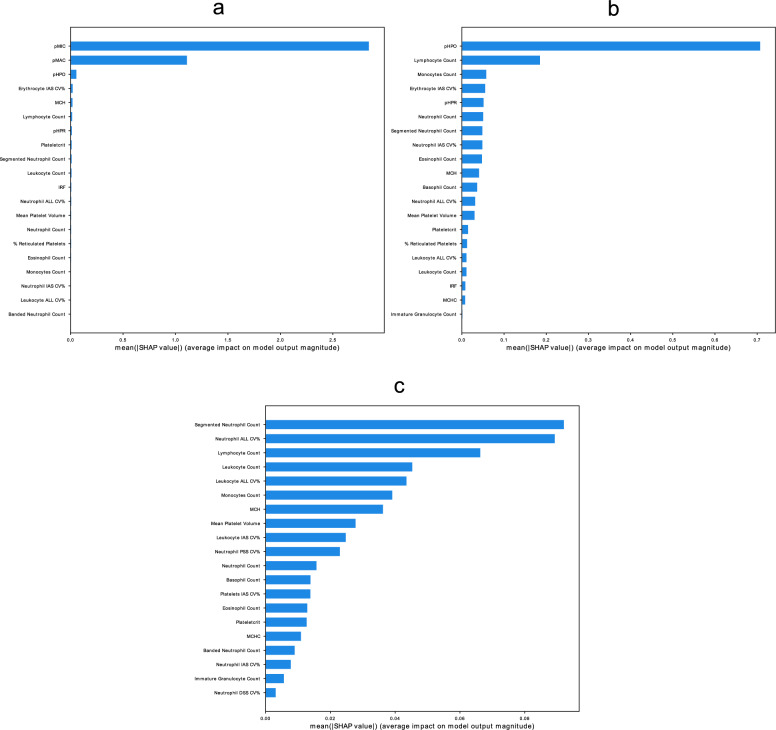
Feature importances for the GB regression models in sets 1, 2 and 3 respectively, showing a large importance for the percentage of microcytic erythrocytes in modelling RDW: (**a**) Feature importance in set 1. (**b**) Feature importance in set 2. (**c**) Feature importances in set 3.

To study the association between blood cell characteristics and RDW, we used all blood cell characteristics as determinants and RDW as outcome. GB regression models showed that the percentage of microcytic and macrocytic erythrocytes were most important in set 1 (RMSE = 0.40, R^2^ = 0.96). pMIC was most important, followed by pMAC, and percentage hypochromic erythrocytes (pHPO—Fig. 2a). In set 2, pHPO showed the highest feature importance , followed by the lymphocyte count, while in set 3 segmented neutrophil count, the CV of the Neutrophil size, and the lymphocyte count showed the highest importances (Fig. 2b and c). However, the models that were used to model RDW with the characteristics of sets 2 and 3, performed considerably worse (RMSE = 1.00, R^2^ = 0.76; RMSE = 1.34 and a R^2^ = 0.57 respectively, Supplementary Fig. 4, Supplementary Table 2). In summary, these models show that RDW is best modelled using erythrocyte size markers, specifically by the percentage of microcytic erythrocyte.

### RDW modelling is not influenced by anemia

In order to further study the explanation of RDW in different subpopulations of anemia, we stratified the cohort into different subgroups and trained our GB models again. Our findings in subgroups of patients with low B12, low folic acid, low iron, and low ferritin, as well as patients with low hemoglobin, high hemoglobin and normal hemoglobin values, did not result in a different outcome compared to the main results. All models showed high feature importances for pMIC, pMAC and to a lesser extent pHPO (Supplementary Table 2).

### In silico validation shows consistent model performance across analyzers and care settings

**Figure 3:**
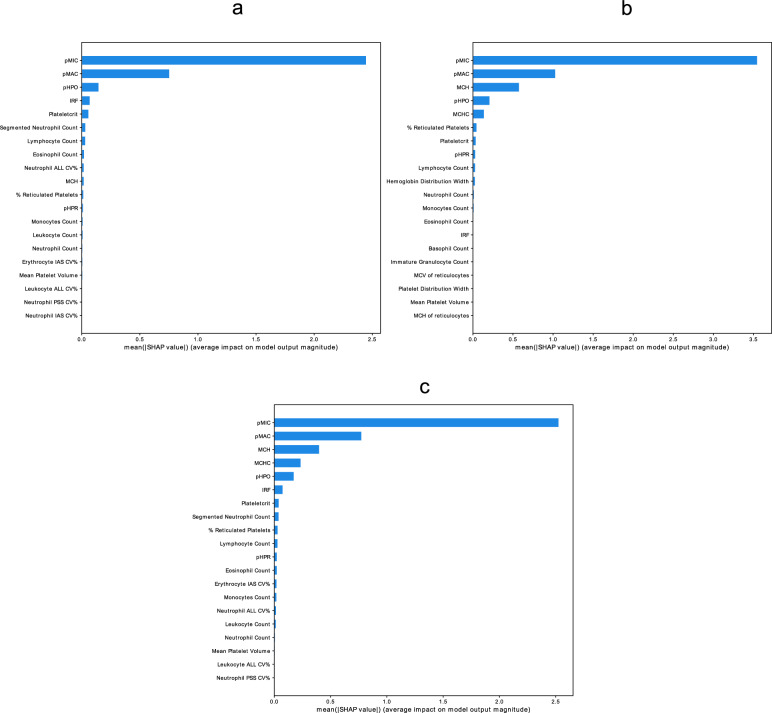
Feature importances in the validation data, using the models predicting RDW showing the high importance of the percentage microcytic erythrocytes in modelling RDW: (**a**) Validation of the feature importances in the Sapphire data from the Alinity comparison study. (**b**) Validation of the feature importances in the Alinity data from the Alinity comparison study. (**c**) Validation of the feature importances in primary and secondary care.

Validation of the model showed that our model, as trained in the original data set, showed high performance, also in the Cell-Dyn Alinity comparison data sets. The RMSE was 0.53 and 0.75 with R^2^ of 0.93 and 0.87 for the Sapphire and Alinity data set respectively. Additionally, calculating feature importances for the models in the validation set showed that pMIC was still highly important, with pMAC as second. pHPO scored lower, and for both sets the MCH scored higher (Fig. 3). The validation in data from primary and secondary care showed that our findings were consistent, with a high model performance (RMSE = 0.46, R^2^ = 0.94). Indeed, the percentage of microcytic erythrocytes was again the most important feature for predicting RDW in these data, followed by pMAC and pHPO (Fig. 3).

### In vitro exposure of erythrocytes to oxidative stress mimics in silico findings

**Figure 4:**
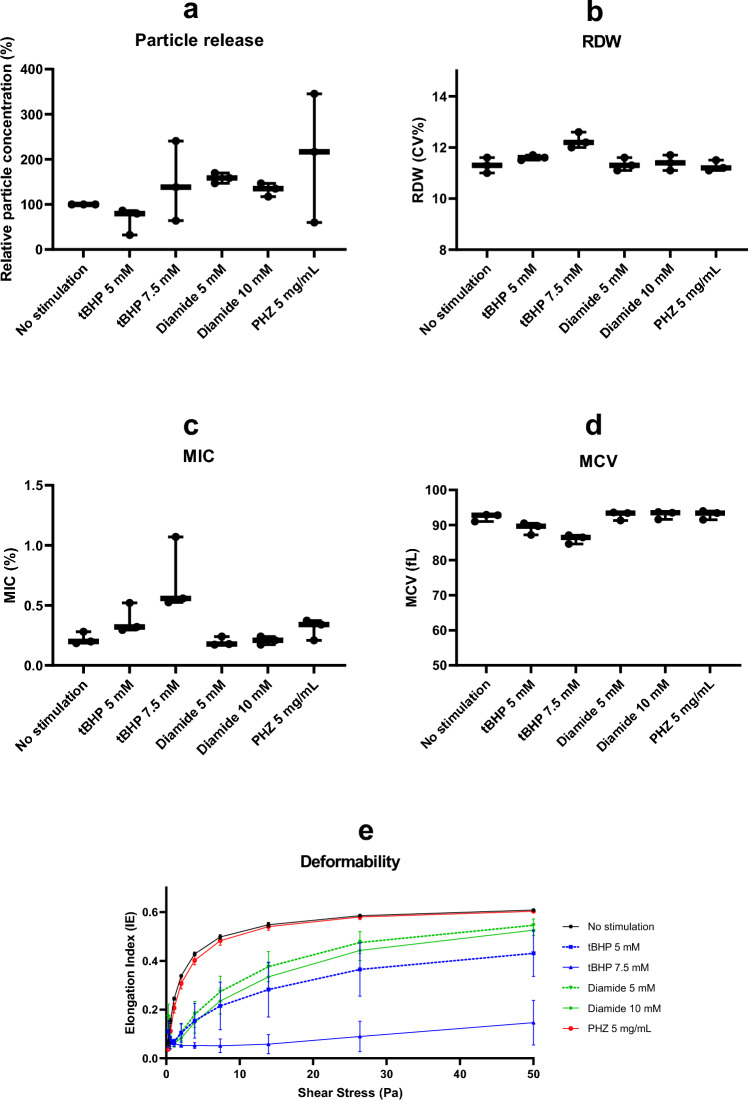
In Vitro results, showing an increase of RDW and microcytic erythrocytes after induction of oxidative stress with tBHP: (**a**) Particle release (vesiculation) did not increase after induction. (**b**) An increase in RDW is shown, (**c**) as well as an increase in the percentage microcytic erythrocytes. (**d**) A decrease in MCV was also seen after induction of oxidative stress. (**e**) Deformability decreased after induction of oxidative stress.

The high importance of pMIC in modelling RDW, and decrease in MCV, were likely not related to iron status or hematopoietic differences, for we found no different results in subgroup analyses. One possible explanation could be that the decrease in erythrocyte size and volume was caused by oxidative stress-induced loss of membrane, a common pathophysiological feature of many diseases^23^. To test this hypothesis, purified erythrocytes were exposed to three commonly used inducers of oxidative stress: tBHP (5 mM and 7.5 mM)^24^, diamide (5 and 10 mM)^25^, and PHZ (5 mg/mL)^26^. Nanoparticle tracking analysis however showed no increase in relative particle concentration compared to baseline for either of the three inducers of oxidative stress (Fig. 4a), for comparison: erythrocytes vesicle release induced by calcium ionophore exposure caused a 500-fold increase in relative particle concentration (data not shown), indicating that these forms of oxidative stress were not associated with an increase in membrane vesiculation. Interestingly however, erythrocytes exposed to tBHP did show a dose-dependent increase in RDW (Fig. 4b) and pMIC (Fig. 4c) as well as a lowered MCV (Fig. 4d). This was accompanied by a dose-dependent decrease in deformability (Fig. 4e). Notably, these changes were not seen upon exposure to diamide and PHZ, indicating that the lipid peroxidation induced by tBHP is responsible for the observed altered erythrocyte features. Hence, we speculate that tBHP-induced lipid peroxidation causes erythrocytes to become smaller and more rigid—explaining the increased RDW and pMIC, thereby inhibiting vesiculation. To test this hypothesis, erythrocytes were treated with Calcium Ionophore to induce vesiculation. As expected, pre-treatment of the cells with tBHP almost entirely inhibited such vesiculation.

## Discussion

In this study, using routine hematologic characteristics of 1,403,663 measurements from 214,315 patients we found that RDW was best modelled using erythrocyte size characteristics, specifically pMIC. We found consistent results in several subgroups of clinical and subclinical anemia and replicated our results across platforms and care settings. Moreover, we did not find any indication for a role of other cell types, for example leukocytes, which could point towards inflammation. We found in vitro evidence that oxidative stress affecting the size of the erythrocytes may play a role in the association between RDW and clinical outcomes.

Our results indicate a large and consistent role for pMIC in modelling RDW. First, our models show the percentage of small erythrocytes is the most important feature associated with increased RDW measurements. Second, we only found small and unstable importances for hematological inflammatory markers, as we found no high importances for leukocyte characteristics, or the characteristics of neutrophils, lymphocytes, monocytes, basophils, and eosinophils.. Third, we also found no different results for anemic patients: hemoglobin values were not among the most important features, and subanalyses in patients without anemia (normal hemoglobin levels) were similar. In subgroup analyses where we hypothesized an importance for macrocytic cells (i.e., low folic acid and vitamin B12 in macrocytic anemia), the feature importance was still highest for pMIC. Additionally, when removing pMIC, pMAC and MCV, model performance dropped dramatically, therefore the ability of the models to reflect the true relationships of hematological parameters and RDW within our data.

Our study is the largest study into modelling RDW using routine hematology markers. Previous studies identified a negative relationship between hemoglobin and RDW which we did observe also^27^. Additionally, a previous study in 250,000 samples found moderate (< 0.7) linear associations between the percentage microcytic and macrocytic erythrocytes and RDW^28^. We used linear (ridge) and non-linear (GB) regression models, and found better performance for the latter, also in subgroup analyses. Because poor performance affects the certainty of the model to reflect the truth, the use of non-linear models in our study provides more insight in the true relationships between hematological characteristics, as these relationships indeed may not always be linear.

In addition, several studies have examined blood levels of biomarkers that are associated with RDW as a first step in unraveling the pathophysiological mechanism of increased RDW^12,13^. RDW could be predicted independently of age, sex, MCV, hemoglobin and ferritin by high-sensitivity C-reactive protein, a biomarker for inflammation, and erythrocyte sedimentation rate^12^. We found no indication of inflammation in our study in the hematological markers for inflammation. Unfortunately we were not able to retrieve C-reactive protein levels for our measurements. Additionally, stratification of RDW has been shown to be associated with several markers for ineffective erythropoiesis, inflammation, and nutritional deficiency^13^.

Subgroup analyses showed no effect of microcytic or macrocytic anemia on the importance of characteristics in modelling RDW. Healthy erythrocytes decrease in volume as they get older, because of budding, where small parts of the membrane are excreted as vesicles. This decrease leads more dense and less deformable cells, and eventual (splenic) clearance from the bloodstream. As we observed that the percentage of microcytic erythrocytes is highly important in explaining RDW, one could speculate that our results are driven by accelerated vesicle budding. In turn, the reason why this vesicle budding is accelerated may be because of increased levels of oxidative stress, often found in various diseases, and is not reflected by blood cell characteristics as measured in this study. The relationship between oxidative stress and accelerated erythrocyte aging has been described elsewhere^29^. However, in set 2 we saw that the percentage of hypochromic cells (pHPO) is an important variable for RDW, rather than MCHC and the percentage hyperchromic cells (pHPR), which would be expected when erythrocytes would be denser after vesicle budding. Additionally, we did not find any evidence for vesicle budding after in vitro induction of oxidative stress. Yet, we did found a decrease in cell volume, as well as an increased RDW and pMIC by tBHP-induced lipid peroxidation. A possible explanation for this phenomenon is that (tBHP-induced) lipid peroxidation causes erythrocytes to become rigid and lose deformability^30^. Hence, we postulate that oxidative stress affecting the size of the erythrocytes may play a role in the association between RDW and clinical outcomes. Of course the relatively short exposure to oxidative stress in vitro represents only a model for (sustained) oxidative stress in vivo, and we can not fully rule out other components that may play a role. However, the knowledge we gained from our in silico research, as well as the results from our in vitro study, do point towards oxidative stress as an important driver, and limited influence for inflammation and anemia.

There are some limitations in this study. First, we used data from routine care settings that do not reflect the general population. We encountered missing data in our subanalyses and although preprocessed with care, we cannot fully rule out the effect of specific diseases that affect blood cell size in our data. For example, we were unable to exclude patients with hemoglobinopathies, though we think it is unlikely this small set of patients significantly influences our results. Using this very large dataset we found consistent results, even in subanalyses, and we are therefore confident that including omitted data will not lead to different results. Additionally, our validation across platforms and health care settings further accentuated our results. Furthermore, feature importances in ensemble methods such as GB can be influenced by collinearity^31^. We corrected for collinearity when filtering the variables, but some variables that were correlated (e.g., neutrophils and white blood cell count) were kept because of biological relevance. pMIC, pMAC, mean corpuscular volume of reticulocytes (MCVr), and MCH highly correlated to MCV, implying that high feature importance values for these variables can mean that MCV might be underrepresented. However, this does not change our finding that erythrocyte characteristics are most important in explaining RDW. Finally, one limitation concerning our in vitro approach is the use of a commonly used yet only a single source of oxidative stress (PHZ) as a model of oxidative stress in vivo. This leaves room for a possible oxidant-dependent effect on hemoglobin oxidation and consequently RDW.

Further research could include scrutinizing the data for specific subgroups of patients or measurements, for example by leveraging data on clinical outcomes, which we were not able to retrieve for this study. For these groups stratified or multilevel analyses could then be performed, to further estimate a population-wide and subgroup effect of hematological markers on RDW. This way, we can further unravel the pathophysiological mechanisms underlying the association between RDW and health.

## Conclusion

In a dataset of 1,403,663 blood sample measurements from routine clinical care, we found that RDW was best modelled by erythrocyte size characteristics, specifically pMIC. Moreover, we did not find any indication for a role of inflammation or anemia, but we found evidence pointing towards decreasing erythrocyte volume, which can be the result of oxidative stress. In this study we have identified important leads for further research on the relationship between RDW and clinical outcomes.

## Supplementary Information

Supplementary Information.

## Data Availability

Data from this study will not be shared publicly considering privacy regulations, but will be made available upon reasonable request.
