# Supplementary material for: In-vitro and in-silico evidence for oxidative stress as drivers for RDW
Source: Sci Rep. 2023 Jun 7;13:9223. doi: 10.1038/s41598-023-36514-5 (PMC10247684; doi:10.1038/s41598-023-36514-5)
Supplement: Supplementary file 1 — Supplementary Information. [file 41598_2023_36514_MOESM1_ESM.docx]

**Supplementary Table 1: Descriptives of all variables that were used**

|  | **1Q** | **median** | **3Q** | **n** | **acronym** |
| --- | --- | --- | --- | --- | --- |
| % Hyperchromic RBC | 0.00 | 0.02 | 0.14 | 860180 | pHPR |
| % Hypohromic RBC | 1.11 | 3.12 | 8.81 | 860180 | pHPO |
| % Macrocytic RBC | 1.01 | 1.79 | 3.39 | 863105 | pMAC |
| % Microcytic RBC | 0.58 | 0.87 | 1.56 | 863105 | pMIC |
| % Reticulated Platelets | 1.70 | 2.45 | 3.65 | 863105 | prP |
| Band Cell Count | 0.00 | 0.00 | 0.00 | 1403663 | bnd |
| Basophil Count | 0.02 | 0.03 | 0.05 | 1403663 | bas |
| Eosinophil Count | 0.06 | 0.12 | 0.23 | 1403663 | eos |
| Hemoglobin | 6.57 | 7.77 | 8.72 | 1403663 | hb |
| Hemoglobin Distribution Width | 6.82 | 7.51 | 8.36 | 860180 | HDW |
| Immature Granulocyte Count | 0.00 | 0.00 | 0.00 | 1403663 | ig |
| Immature Reticulocyte Fraction | 0.23 | 0.30 | 0.39 | 1102508 | irf |
| Lymphocyte ALL CV% | 3.54 | 4.62 | 5.62 | 1401810 | Lacv |
| Lymphocyte ALL Mean | 95.47 | 99.15 | 102.52 | 1401810 | Lamn |
| Lymphocyte Count | 1.10 | 1.61 | 2.18 | 1403663 | lym |
| Lymphocyte IAS CV% | 3.87 | 4.51 | 5.21 | 1401890 | Licv |
| Lymphocyte IAS Mean | 73.40 | 75.45 | 77.56 | 1401890 | Limn |
| Mean Corposcular Hemoglobin | 1.81 | 1.90 | 1.98 | 1403663 | MCH |
| Mean Corposcular Hemoglobin Concentration | 0.20 | 0.21 | 0.22 | 1403663 | MCHC |
| Mean Corposcular Hemoglobin Concentration in Reticulocytes | 28.11 | 29.40 | 30.53 | 860180 | MCHCr |
| Mean Corposcular Hemoglobin in Reticulocytes | 27.88 | 30.02 | 31.62 | 860180 | MCHr |
| Mean Corposcular Volume | 86.82 | 90.15 | 93.58 | 1403663 | MCV |
| Mean Corposcular Volume in Reticulocytes | 95.63 | 100.12 | 104.65 | 860180 | MCVr |
| Mean Platelet Volume | 7.06 | 7.72 | 8.52 | 1403663 | mpv |
| Monocyte Count | 0.47 | 0.63 | 0.85 | 1403663 | mon |
| Neutrophil ALL CV% | 2.28 | 2.55 | 2.92 | 1400983 | nacv |
| Neutrophil ALL Mean | 141.35 | 148.52 | 154.97 | 1400983 | namn |
| Neutrophil Count | 3.95 | 5.76 | 8.67 | 1403663 | neu |
| Neutrophil DSS CV% | 14.56 | 15.50 | 16.41 | 1400983 | ndcv |
| Neutrophil DSS Mean | 25.03 | 27.51 | 30.06 | 1400983 | ndmn |
| Neutrophil FL3 CV% | 7.11 | 8.11 | 8.99 | 1400975 | nfcv |
| Neutrophil FL3 Mean | 68.49 | 70.19 | 72.06 | 1400975 | nfmn |
| Neutrophil IAS CV% | 3.22 | 3.53 | 3.85 | 1400967 | nicv |
| Neutrophil PSS CV% | 6.67 | 8.49 | 9.80 | 1400926 | npcv |
| Neutrophil PSS Mean | 114.31 | 122.62 | 130.65 | 1400926 | npmn |
| Neutrophill IAS Mean | 130.27 | 134.69 | 139.35 | 1400967 | nimn |
| Platelet IAS CV% | 16.50 | 17.14 | 17.83 | 1403663 | Picv |
| Platelet IAS Mean | 140.44 | 144.55 | 148.68 | 1403663 | Pimn |
| Platelet PSS CV% | 12.81 | 13.49 | 14.37 | 1403663 | Ppcv |
| Platelet PSS Mean | 121.68 | 124.87 | 128.14 | 1403663 | ppmn |
| Platelet Volume Distribution Width | 15.70 | 16.12 | 16.58 | 1403663 | pdw |
| Plateletcrit | 0.15 | 0.19 | 0.24 | 1403663 | pct |
| RBC FL3 CV% | 9.86 | 11.23 | 12.83 | 1025390 | rbcfcv |
| RBC FL3 Mean | 81.32 | 83.76 | 86.17 | 1025390 | rbcfmn |
| RBC IAS CV% | 1.53 | 1.67 | 1.84 | 1025390 | rbcicv |
| RBC IAS Mean | 179.89 | 181.69 | 183.26 | 1025390 | rbcimn |
| Red Blood Cell Distribution Width | 11.84 | 12.62 | 14.06 | 1403663 | RDW |
| Reticulocyte Count | 51.18 | 68.07 | 90.59 | 1102508 | retc |
| Segmented Neutrophil Count | 3.91 | 5.68 | 8.45 | 1403663 | seg |
| WBC Viability Factor | 0.99 | 0.99 | 1.00 | 1403663 | wvf |
| White Blood Cell Count | 6.50 | 8.53 | 11.50 | 1403663 | WBC |

**Supplementary Fig 1: correlation matrix of variables in the unfiltered data set**

**
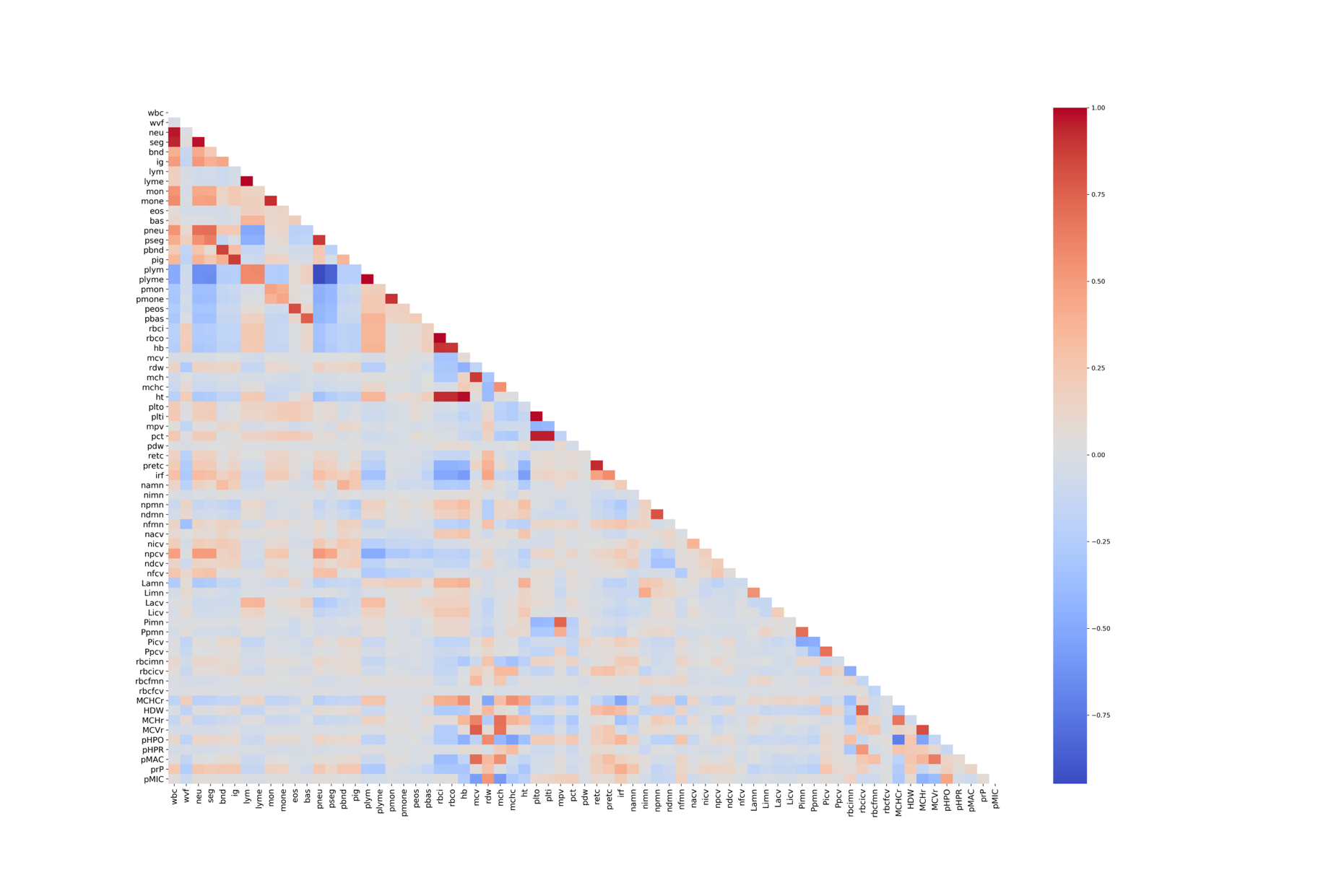
**

*Overview of the correlations between variables in the data. Acronyms are described in the SDC, Table 1. Dark red means high positive correlation, dark blue means high negative correlation*

**Supplementary Fig 2: Model variables correlation matrix**


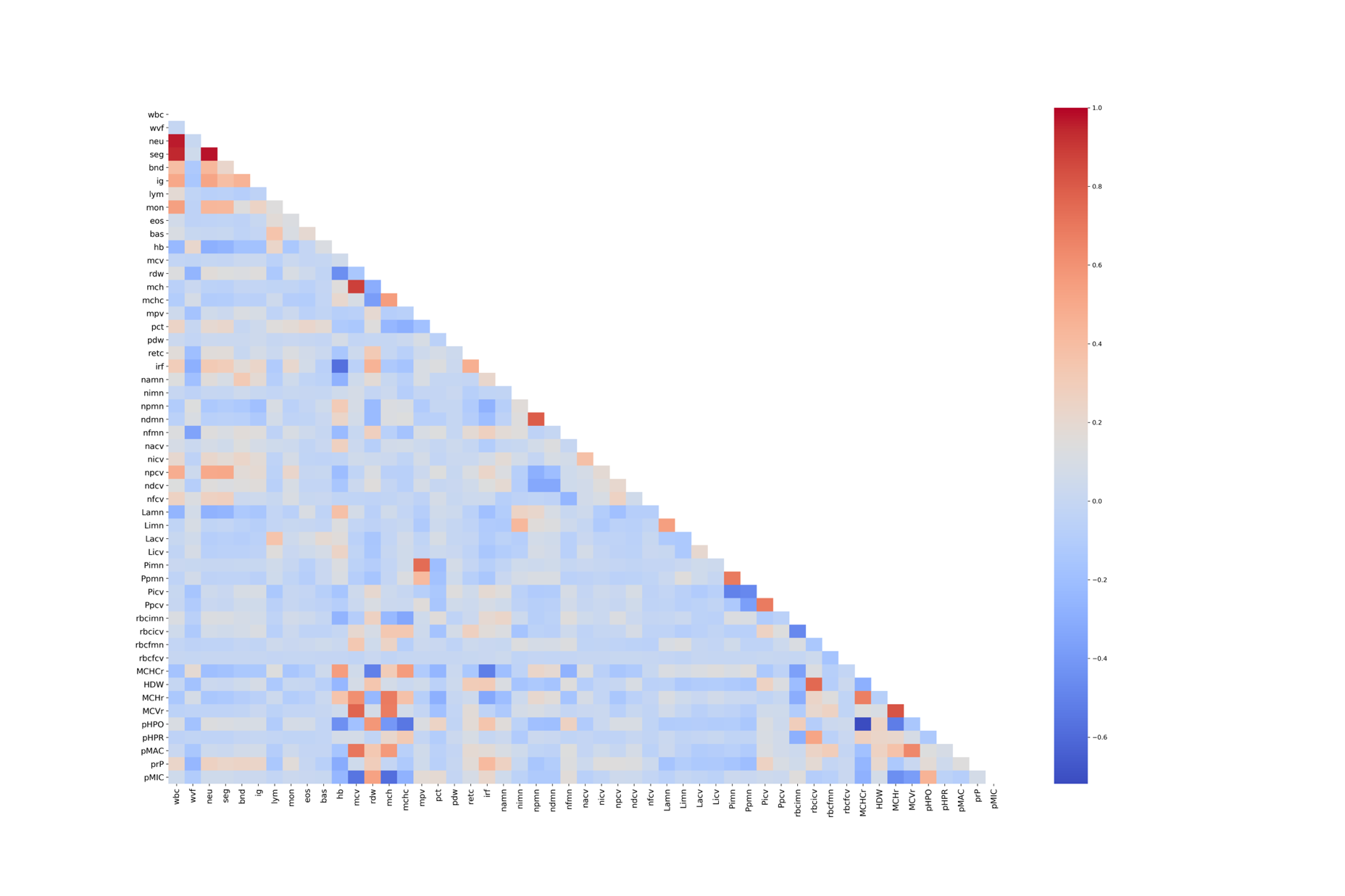


*Overview of the correlations between variables in the data used for modelling. Acronyms are described in the SDC, Table 1. Dark red means high positive correlation, dark blue means high negative correlation*

**Supplementary Fig 3: Plot showing the increase of RDW over age, stratified according to sex**


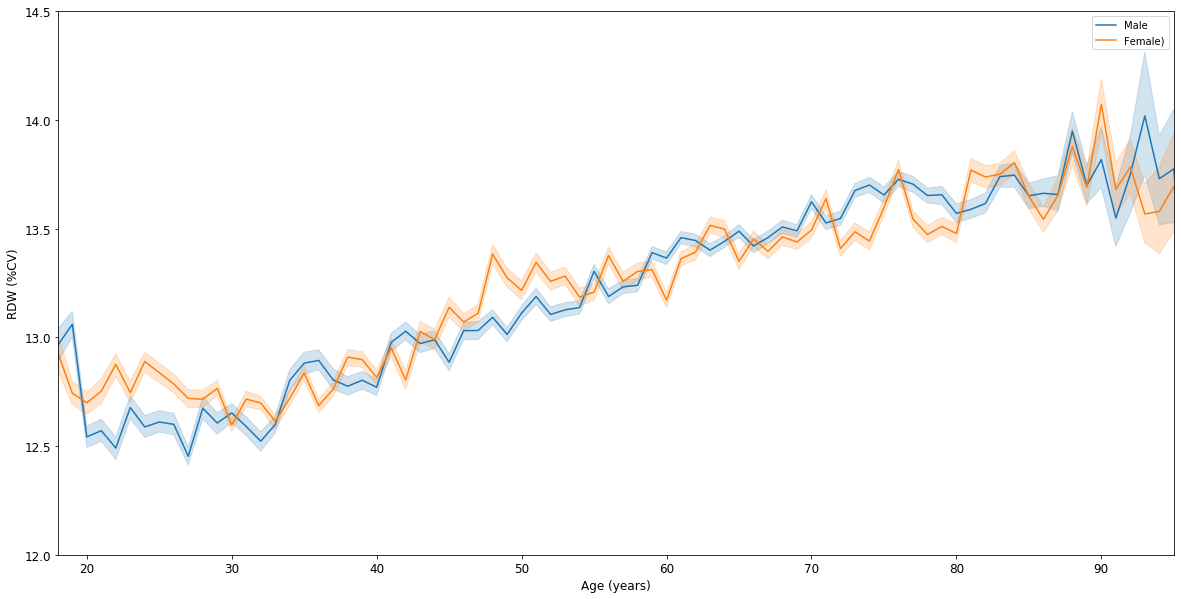


**Supplementary Fig 4: Performance Gradient Boosting Regression**


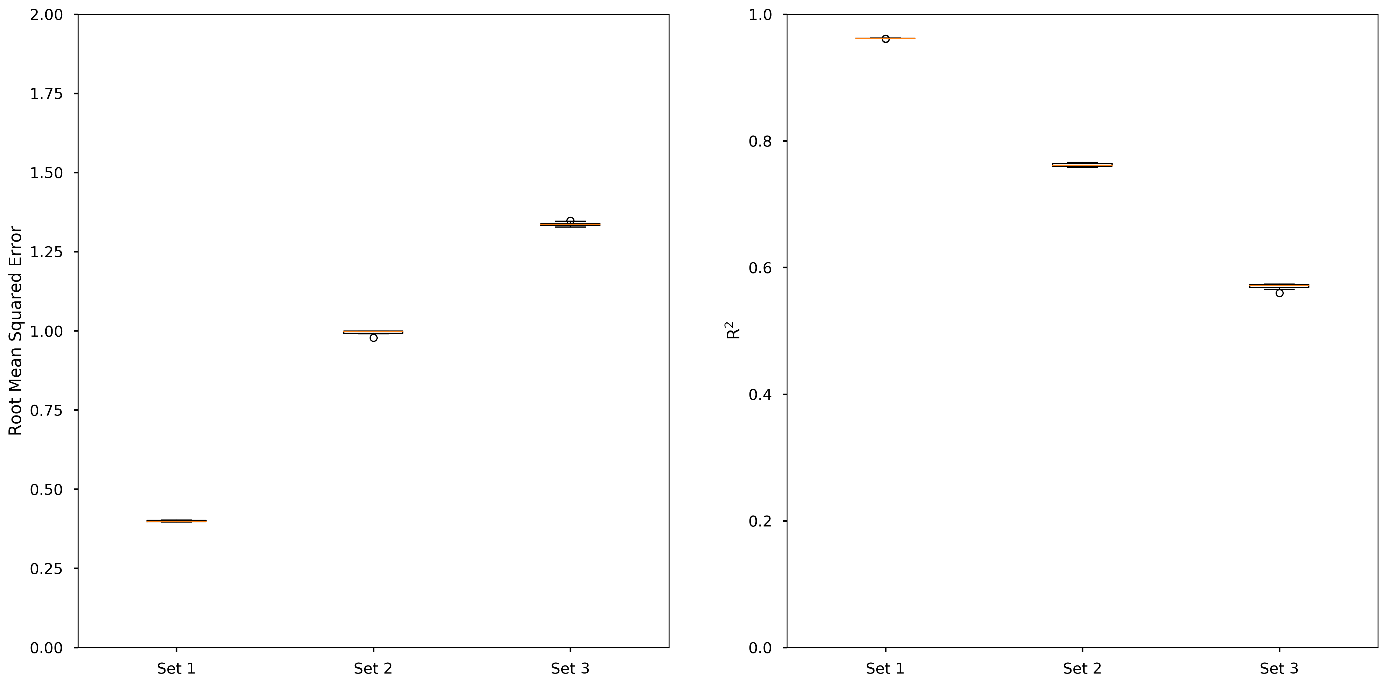


*Root Mean-Squared Error and R^2^ score across the different sets for the Gradient Boosting regression showing an increase in error and decrease in R^2^ across sets 1 to 3*

**Supplementary Table 2: performances of subgroup analyses and the three most important features, N is the amount of samples for these models.**

|  | **N** | **Set 1** | | | **Set 2** | | | **Set 3** | | |
| --- | --- | --- | --- | --- | --- | --- | --- | --- | --- | --- |
|  |  | **RMSE** | **R2** | **Shapley** | **RMSE** | **R2** | **Shapley** | **RMSE** | **R2** | **Shapley** |
|  |  | 0.40 | 0.96 | pMIC: 2.918 | 1.00 | 0.76 | pHPO: 0.870 | 1.34 | 0.57 | Seg: 0.104 |
| Global model | 858,137 |  |  | pMAC: 1.136 |  |  | Lym: 0.139 |  |  | Neu: 0.070 |
|  |  |  |  | pHPO: 0.054 |  |  | Nicv: 0.107 |  |  | Lym: 0.040 |
|  |  | 0.58 | 0.92 | pMIC: 2.167 | 1.19 | 0.65 | pHPO: 0.363 | 1.46 | 0.47 | Lym: 0.026 |
| Low Ferritin | 11,261 |  |  | pMAC: 0.282 |  |  | Lym: 0.050 |  |  | PCT: 0.024 |
|  |  |  |  | pHPO: 0.084 |  |  | pHPR: 0.044 |  |  | Mon: 0.013 |
|  |  | 0.56 | 0.92 | pMIC: 2.966 | 1.18 | 0.66 | pHPO: 0.207 | 1.47 | 0.46 | Lym: 0.02 |
| Low Iron | 16,581 |  |  | pMAC: 0.558 |  |  | irf: 0.085 |  |  | Eos: 0.013 |
|  |  |  |  | MCH: 0.069 |  |  | MCH: 0.068 |  |  | MCHC: 0.013 |
|  |  | 0.69 | 0.88 | pMIC: 1.250 | 1.29 | 0.58 | pHPO: 0.305 | 1.53 | 0.41 | Lym: 0.304 |
| Low Vit B12 | 3623 |  |  | pMAC: 1.140 |  |  | Bas: 0.019 |  |  | Mon: 0.038 |
|  |  |  |  | pHPO: 0.204 |  |  | Mon: 0.018 |  |  | PCT: 0.022 |
| Low Folic Acid |  | 0.59 | 0.92 | pMIC: 1.960 | 1.25 | 0.62 | pHPO: 0.585 | 1.51 | 0.46 | Lym: 0.033 |
|  | 7172 |  |  | pMAC: 0.858 |  |  | Lym: 0.036 |  |  | WBC: 0.029 |
|  |  |  |  | pHPO: 0.084 |  |  | irf: 0.027 |  |  | Mon: 0.026 |
|  |  | 0.41 | 0.95 | pMIC: 2.54 | 0.91 | 0.76 | pHPO: 0.728 | 1.22 | 0.59 | Lym: 0.073 |
| Patients < 40 | 199468 |  |  | pMAC 1.053 |  |  | NACV: 0.078 |  |  | MCH: 0.022 |
|  |  |  |  | pHPO: 0.038 |  |  | Seg: 0.066 |  |  | LICV: 0.021 |
|  |  | 0.40 | 0.96 | pMIC: 2.469 | 1.02 | 0.76 | pHPO: 0.867 | 1.35 | 0.57 | MCH: 0.068 |
| People ≥ 40 | 658,669 |  |  | pMAC : 1.256 |  |  | Lym: 0.182 |  |  | Lym: 0.048 |
|  |  |  |  | Lym: 0.108 |  |  | pHPR: 0.100 |  |  | Bas: 0.032 |
|  |  | 0.41 | 0.95 | pMIC: 3.699 | 0.91 | 0.76 | pHPO: 0.442 | 1.22 | 0.59 | LACV: 0.114 |
| Women | 417,253 |  |  | pMAC: 1.452 |  |  | MCH: 0.072 |  |  | Mon: 0.049 |
|  |  |  |  | Neu: 0.029 |  |  | pHPR: 0.059 |  |  | NACV: 0.049 |
|  |  | 0.39 | 0.96 | pMIC: 2.081 | 0.96 | 0.77 | pHPO: 0.442 | 1.28 | 0.59 | LACV: 0.120 |
| Men | 440,884 |  |  | pMAC: 0.665 |  |  | MCH: 0.072 |  |  | MCH: 0.075 |
|  |  |  |  | pHPO: 0.027 |  |  | pHPR: 0.059 |  |  | LICV: 0.053 |
|  |  | 0.45 | 0.96 | pMIC: 3.737 | 1.17 | 0.74 | lym: 0.259 | 1.56 | 0.53 | MPV: 0.049 |
| Low HB | 440,103 |  |  | pMAC: 1.218 |  |  | pHPR: 0.121 |  |  | Bas: 0.042 |
|  |  |  |  | pHPO: 0.038 |  |  | pHPO: 0.107 |  |  | LACV: 0.042 |
|  |  | 0.43 | 0.96 | pMIC: 3.329 | 1.08 | 0.75 | pHPO: 0.245 | 1.42 | 0.55 | Lym: 0.098 |
| Sub Clinical HB | 677,496 |  |  | pMAC: 1.345 |  |  | Lym: 0.232 |  |  | MPV: 0.082 |
|  |  |  |  | pHPO: 0.062 |  |  | NICV: 0.071 |  |  | LICV: 0.072 |
|  |  | 0.35 | 0.93 | pMAC: 0.983 | 0.78 | 0.65 | pHPO: 0.526 | 1.01 | 0.41 | Lym: 0.064 |
| Normal HB | 406,037 |  |  | pMIC: 0.090 |  |  | RBCICV: 0.038 |  |  | NPCV: 0.024 |
|  |  |  |  | pHPR: 0.038 |  |  | IRF: 0.036 |  |  | Mon: 0.023 |
|  |  | 0.34 | 0.90 | pMIC: 0.438 | 0.70 | 0.59 | pHPO: 0.189 | 0.87 | 0.37 | Mon: 0.043 |
| High HB | 144,062 |  |  | pMAC: 0.229 |  |  | MCH: 0.071 |  |  | Lym: 0.019 |
|  |  |  |  | PCT: 0.014 |  |  | pHPR: 0.024 |  |  | PCT: 0.012 |
